# The Per-Ingvar Brånemark Era (1929–2014): Evolution of a No Compromise Prosthetic Dental Replacement

**DOI:** 10.7759/cureus.71708

**Published:** 2024-10-17

**Authors:** Sumeet Agarwal, Laresh Mistry, Saloni Mistry, Ishan Kadam, Shefali Bhiwapurkar, Shefali Talekar, Saba Kondkari

**Affiliations:** 1 Department of Prosthodontics, Bharati Vidyapeeth (Deemed to be University) Dental College and Hospital, Navi Mumbai, IND; 2 Department of Pediatric and Preventive Dentistry, Bharati Vidyapeeth (Deemed to be University) Dental College and Hospital, Navi Mumbai, IND; 3 Department of Prosthodontics, Dr. G.D. Pol Foundation's Y.M.T. Dental College and Hospital, Navi Mumbai, IND; 4 Department of Conservative Dentistry and Endodontics, Bharati Vidyapeeth (Deemed to be University) Dental College and Hospital, Navi Mumbai, IND; 5 Department of Dentistry, Bharati Vidyapeeth (Deemed to be University) Dental College and Hospital, Navi Mumbai, IND

**Keywords:** brånemark, historical vignette, implants, modern dentistry, osseointegration

## Abstract

Per-Ingvar Brånemark (1929-2014) revolutionized 21st-century dentistry with the introduction of osseointegration, and his invention of dental implants revolutionized the restoration of smiles all over the world. His accidental discovery that titanium bonds with bone in the 1950s revolutionized restorative dentistry, especially for prosthetic teeth caused by trauma or disease. Through the course of decades, his work was translated into clinical techniques and materials that have helped millions to reacquire oral function. His commitment to research, clinical application, and global education earned him the title of the father of modern dental implantology, with his influence still being felt in introducing millions of fellow clinicians into the field. This is the narrative of Brånemark's life and career, the main point being some of his outstanding achievements in the dental field, including the introduction of titanium implants in 1965 and osseointegration spreading around the globe in the 1980s.

## Introduction and background

Dr. Per-Ingvar Brånemark was born on May 3, 1929, in Sweden. While growing up in his family, which put a great value on education, his interest in science and medicine caught him early on [1]. In 1948, he went to the University of Lund, where he initially began studies in anatomy and physiology [2]. His interest in bone physiology in his early career was a leading factor to a milestone moment in his life, in the 1950s, when he discovered the unique characteristics of titanium as a biocompatible material suited for bone healing. This work spawned his contributions to the world of dentistry, particularly involving the creation of dental implants. He eventually became a lecturer of anatomy at Gothenburg University and lab head from 1963 to 1977. The renowned man died suddenly in Gothenburg, Sweden on 20th December 2014, aged 85, from cardiac arrest [2]. We pay homage to the esteemed scholar who had impressive input in the medical fraternity.

It takes an understanding of and use of both biological and mechanical principles to successfully integrate periodontal and restorative dentistry for both natural teeth and implants. An appropriate band of connected gingiva can minimize patient discomfort in areas of cosmetic concern, lower the likelihood of gingival recession during tooth preparation, and streamline restorative operations [3].

Prosthetics have gone a long way from the days of easily sliding and uncomfortable removable dentures and simple bridges. Sadly, these devices were typically affixed to nearby teeth, which frequently compromised the integrity of those teeth, and they were ill-suited for the best functional or aesthetic outcomes [4]. Instead of being enclosed within a denture, the bar joint technology offered anchorage for full fixed prostheses with functioning similar to normal mastication. Dental implants are an innovative move taken in restorative dentistry since this switch from detachable to fixed solutions not only improved dental appearances but also restored the oral health of patients that they had been too afraid to reveal [5].

## Review

The discovery of osseointegration

Dr. Brånemark was performing his research at the University of Lund when he had an unexpected event happen in 1952 that would ultimately revolutionize modern dental care. The major breakthrough of 1952 by Brånemark occurred purely coincidentally [6]. Brånemark was studying bone healing and blood circulation, so he inserted a titanium chamber into the leg of a rabbit to observe blood flow through a microscope. Months later, he attempted to remove the chamber, and behold, the titanium had bonded to the bone. This event was significant because it represented the first instance when osseointegration or bone tissue connecting to a non-biological component, in this case, titanium, was observed. Osseointegration, a term coined by Brånemark, refers to the formation of a direct structural and functional connection between ordered, living bone and the surface of a load-carrying implant [7]. His interest led him to further investigate the clinical significance of this phenomenon, particularly in dental applications. The continuous research of the subsequent decades by Brånemark came out as a contradiction from these facts, confirming titanium as an essential material for implant dentistry technology [8-10].

The first dental implant and clinical trials

Brånemark's first successful dental implant took place in 1965 when he inserted titanium implants in a patient who had complete edentulism and later regained oral function. This was the first milestone in dental implantology where osseointegration was first applied in humans, and the results were groundbreaking [10]. For many years after that, Brånemark as well as members of his team from the University of Gothenburg conducted huge clinical testing to determine whether titanium implants are safe and successful. By 1977, Brånemark had acquired sufficient clinical evidence to publish his results in which he reported that these titanium implants could fuse reliably with bone and thus could offer a stable base for dental prosthetics [10,11].

Overcoming challenges and building credibility

The early years of Brånemark's work in dental implants were not very open-minded. In 1977, Brånemark established the Institute of Applied Biotechnology in Gothenburg, which served as a principal hub of excellence for pioneering studies in implant design, bone biology, and clinical care. The early medical knowledge indicated that the human body was generally aggressive toward foreign materials, as they caused inflammation, which drew skepticism on Brånemark's osseointegration studies. However, many in the clinical field - be it medicine or dentistry - were concerned about long-term clinical effectiveness due to the fear of complications from infection and implant failures. Brånemark did not deter from the concern and continued with more rigorous scientific validation. Through clinical trials and longitudinal studies, he proved that the success rate for titanium implants was achieved at 90% or higher even after 10 years of use [12]. This persistence paid off when, in the 1970s, the dental community began to realize the sheer potential of osseointegration [13].

Global adoption and commercialization

Brånemark's discoveries were not widely accepted in the dental community until the early 1980s. The pioneers of dentistry were once united in the first international conference on osseointegration in 1981 leading dentists and researchers from around the world gathered to share research. After this conference, it was unquestionably established that osseointegration is a predictable and successful method to rehabilitate oral functions [14]. The recognition of Brånemark's work was further promoted in 1982 when the Brånemark System was introduced to the market by Nobel Biocare, a Swedish company that widely made his titanium implants accessible for use to dentists all over the world [13]. These were Brånemark-type dental implants and by the late 1980s, it became an international standard applied millions of times, improving the quality of life of countless patients.

Impact on modern dentistry

Brånemark's discovery has giant implications. Osseointegration not only established a cure for tooth loss but also opened the door forward in maxillofacial prosthetics and reconstructive surgery. By the 1990s, his techniques had been adopted not only in dentistry but also in orthopedic surgery, especially with regard to joint replacements and limb prosthetics [14]. His work has opened new horizons for the treatment of patients who, previously, had very few possibilities for dental rehabilitation, such as those treated for traumatic injuries, congenital anomalies, and severe periodontal diseases. Brånemark's techniques have also brought a revolution in the aesthetic aspect of dentistry since they opened up more natural-looking and permanent solutions than traditional dentures [7,14].

The Brånemark Osseointegration Center

In 1989, the Brånemark Osseointegration Center was formed as a specialized clinic in Gothenburg, Sweden. This clinic has been one step ahead of all others in establishing dental implant restoration as a routine clinical practice technique. After the success of dental implant reconstruction, the clinic branched into orthopedics, giving the option of replacing either small or major joints. For degenerative osteoarthritis disease, the clinic started with small joints, such as the interphalangeal joints of the hand, and moved on to replacing damaged knees, elbows, and hips. This advancement greatly enhanced the effectiveness of rehabilitation with prosthetic members. Conceptions of oral implantology have been duly utilized for bone-supported hearing aids and maxillofacial rehabilitation. Patients feel much more confident while using their prostheses and while interacting with others. In addition, complicated orofacial abnormalities have been adequately addressed with implants by virtue of better surgical techniques [2,15].

A mini-Nobel

He was awarded the much coveted "Swedish Society of Medicine's Soderberg Prize," popularly referred to as the "Mini-Nobel" in 1992. He was also rewarded with a distinguished medal for technical achievement by the Swedish Engineering Academy. He was awarded a particular medal by the Harvard School of Dental Medicine for his contribution to the field of dental implants [2].

Development of zygomatic implants

He found zygomatic implants in 1998, which are for patients who have advanced bone loss within the upper jaw. These implants attach to the zygomatic bone in cases where there is a shortage of bone for the traditional dental implant and thereby avoid grafting. This innovation brought forth dental implants to the treatment possibilities for a wider number of patients, considering that many patients were left with few alternatives because of compromised bone structures [16]. This significant achievement effectively further solidified him in the history books as a visionary concerning the future of restorative dentistry [11,14,16].

Innovations in implantology

Brånemark's studies resulted in significant designs and applications of dental implants. He then manufactured several systems designed for specific clinical situations, such as single-tooth replacement, full-arch prostheses, and zygomatic implants for extreme bone loss patients. His innovations gave the patient a more stable, functional, and aesthetic approach to edentulism [14].

Implant over fixed and removable prosthesis

When it comes to types of prosthesis, earlier forms included removable dentures and basic bridges that were uncomfortable and could easily slide. Unfortunately, these devices were generally attached to neighboring teeth, often compromising the integrity of such teeth and they were not well suited for best aesthetic or functional results [4]. The bar joint technology provided anchorage for complete fixed prostheses with functionality like natural mastication rather than within a denture. This change from removable to fixed solutions not only enhanced dental aesthetics but also restored the oral health of patients that they have been too scared to show, which is why dental implants are an innovative step taken in restorative dentistry [5].

There is an abundance of advantages over fixed partial dentures as they eliminate the need for preparation of the abutment tooth hence being conservative, maintaining the health of bone, having a natural appearance and feel, helping maintain easy oral hygiene, and are very long-lasting [16].

Later career and legacy

His contributions to dentistry and medical science earned him many accolades throughout his life. In 2001, Brånemark entered the National Inventors Hall of Fame, which further amplified his legacy as one of the greatest figures in modern medicine and dentistry [17]. The Doctor of Dental Science degree was received by Brånemark on December 19, 2003. The degree was conferred by the Honorable Justice Kim Santow, the Chancellor. This was in recognition by the University Senate whose resolution on April 8, 2002, granted him the degree of Doctor of Dental Science (honoris causa). He remained a strong proponent of interdisciplinarity even until the last years of his life. He felt that the future of implantology and prosthetics lay with the integration of bio-medical, engineering, and clinical research. Brånemark's work has impacted generations of dental professionals whose techniques form a foundational part of dental education worldwide [18]. His work went on to influence the world of dental research and practice long after his death in the 21st century, as implantology is constantly evolving and is based directly on the work that he started [17]. During his lifetime, by the time he died in 2014, Brånemark had already written more than 1,000 scientific papers and taught hundreds of clinicians and researchers who were involved with the practice of dental implantology [19].

**Figure 1:**
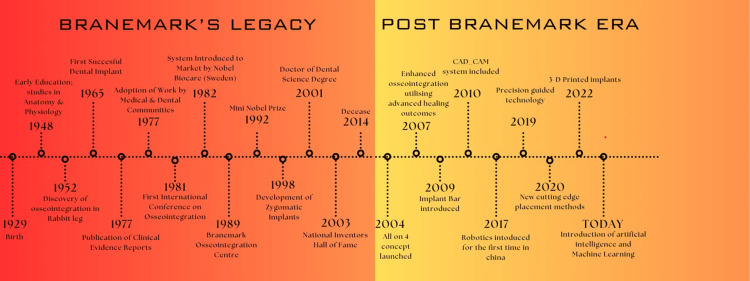
Summary of Per-Ingvar Brånemark’s contribution to the medical field. Figure created by author Saba Kondkari, and checked and verified by authors Sumeet Agarwal and Laresh Mistry.

Figure 1 illustrates a summary of Per-Ingvar Brånemark’s contribution to the medical field.

## Conclusions

Per-Ingvar Brånemark discovered osseointegration, which later took top positions in medicine and dentistry. His discovery of osseointegration started with an everyday observation in the leg of a rabbit and later had tremendous implications for millions of patients worldwide. His research and clinical trials, in association with collaboration with industry, changed the face of dental prosthetics as well because dental implants became commonplace and a very reliable treatment for patients suffering from edentulism. With his relentless pursuit of excellent treatment for patients, he revolutionized the approach of dental professionals in treating tooth loss and its restoration. Today, Brånemark's principle of osseointegration-based dental implants is taken to be the gold standard for permanent tooth replacement. His legacy does not just lie in the millions of patients who have been treated based on his innovations but also in the continued advancements in dental materials and techniques inspired by his work. Brånemark's contributions will forever be foundational to the future of restorative dentistry.
